# Synergistic effects of the invasive Chinese tallow (*Triadica sebifera*) and climate change on aquatic amphibian survival

**DOI:** 10.1002/ece3.857

**Published:** 2013-11-05

**Authors:** Daniel Saenz, Erin M Fucik, Matthew A Kwiatkowski

**Affiliations:** 1 Southern Research Station, U.S. Department of Agriculture, Forest Service506 Hayter Street, Nacogdoches, Texas, 75965, USA; 2 Department of Biology, Stephen F. Austin State UniversityP.O. Box 13003, Nacogdoches, Texas, 75962, USA

**Keywords:** Amphibians, breeding phenology, Chinese tallow, climate change, invasive plant, weather

## Abstract

Changes in climate and the introduction of invasive species are two major stressors to amphibians, although little is known about the interaction between these two factors with regard to impacts on amphibians. We focused our study on an invasive tree species, the Chinese tallow (*Triadica sebifera*), that annually sheds its leaves and produces leaf litter that is known to negatively impact aquatic amphibian survival. The purpose of our research was to determine whether the timing of leaf fall from Chinese tallow and the timing of amphibian breeding (determined by weather) influence survival of amphibian larvae. We simulated a range of winter weather scenarios, ranging from cold to warm, by altering the relative timing of when leaf litter and amphibian larvae were introduced into aquatic mesocosms. Our results indicate that amphibian larvae survival was greatly affected by the length of time Chinese tallow leaf litter decomposes in water prior to the introduction of the larvae. Larvae in treatments simulating warm winters (early amphibian breeding) were introduced to the mesocosms early in the aquatic decomposition process of the leaf litter and had significantly lower survival compared with cold winters (late amphibian breeding), likely due to significantly lower dissolved oxygen levels. Shifts to earlier breeding phenology, linked to warming climate, have already been observed in many amphibian taxa, and with most climate models predicting a significant warming trend over the next century, the trend toward earlier breeding should continue if not increase. Our results strongly suggest that a warming climate can interact with the effects of invasive plant species, in ways we have not previously considered, to reduce the survival of an already declining group of organisms.

## Introduction

Shifts in climate can have negative effects on populations of various organisms, including amphibians (Beebee 1995; Gibbs and Breisch 2001; Parmesan and Yohe 2003; Blaustein et al. 2010). Temperature and precipitation can influence an amphibians' life cycle, particularly breeding activities (Gosner and Black 1955; Busby and Brecheisen 1997; Donnelly and Crump 1998; Saenz et al. 2006). Most North American amphibians lay eggs in water and the amount and timing of precipitation can affect amphibian reproductive activities and yearly reproductive output of an amphibian population (Conant and Collins 1998; Saenz et al. 2006). Water availability also affects adult amphibians due to water loss from skin and respiratory systems (Carey and Alexander 2003).

Warming climates influence body condition, physiological processes, and larval development of some amphibians (Reading 1998, 2007; Walther et al. 2002; Corn 2005). Other consequences of climate warming are changes in range and distribution of species and changes in interactions between species (Collatz et al. 1998; Blaustein et al. 2001; Walther et al. 2002; Badeck et al. 2004). Parmesan and Yohe (2003) estimated that over half (59%) of 1598 plant and animal species documented had experienced measurable changes in phenology and/or distribution over the past 20–140 years due to shifts in climate. Amphibian spring phenology advancement is twice as strong as that of trees, birds, and butterflies, and nearly eight times stronger than herbs, grasses, and shrubs (Parmesan 2007). In temperate regions, increases in temperature can initiate amphibian emergence from hibernation and influence reproductive activities (Gibbs and Breisch 2001; Oseen and Wassersug 2002; Carey and Alexander 2003; Saenz et al. 2006).

Some amphibian declines have been attributed to the establishment of invasive animal and plant species (Doubledee et al. 2003; Brooks et al. 2004; Brown et al. 2006). Introduced trout (*Salmo trutta* and *Onchorhynchus* spp.) and mosquito fish (*Gambusia* spp.), in the United States and Australia, respectively, induce behavioral changes such as delayed metamorphosis in native amphibians and a reduction or elimination in amphibian populations through direct predation (Gillespie 2001; Knapp and Matthews 2000; Lawler et al. 1999; Morgan and Buttemer 1996; Pyke and White 2000). Invasive crayfish (*Procambarus clarki*) reduced the breeding success of California newts (*Taricha torosa*) in southern California mountain streams (Gamradt and Kats 1996) and invasive earthworms appear to be causing declines in woodland salamander abundance by degrading foraging habitat in Pennsylvania and New York (Maerz et al. 2009). Even introduced invasive frog species, such as the Cuban treefrog (*Osteopilus septentrionalis*), the cane toad (*Bufo marinus*), and American bullfrog (*Rana catesbeiana*), are detrimental to native amphibians (Crossland 2000; Hayes and Jennings 1986; Moyle 1973; Smith 2005).

Invasive plant species can have detrimental effects on amphibian populations by altering chemical and physical habitat features, influencing decomposition and nutrient dynamics and altering the trophic structure of invaded ecosystems (Brooks et al. 2004; Brown et al. 2006; Maerz et al. 2005a, 2010; Watling et al. 2011). Maerz et al. (2005b) suggested that invasive Japanese knotweed (*Fallopia japonica*) indirectly reduced foraging by frogs by reducing arthropod abundance. Amphibian larvae exposed to invasive plants in aquatic environments, such as purple loosestrife (*Lythrum salicaria*) and Amur honeysuckle (*Lonicera maackii*), have shown reduced survival compared with exposure to native plants, likely due to direct and indirect effects of phytochemicals negatively acting on tadpoles (Maerz et al. 2005b; Brown et al. 2006; Watling et al. 2011).

**Figure 1:**
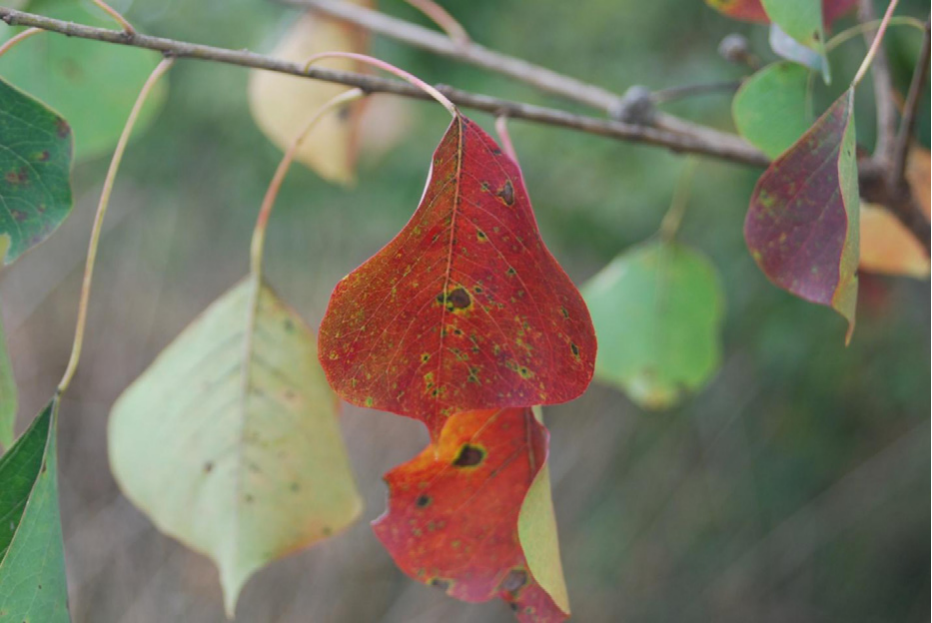
Chinese tallow (Triadica sebifera) is an invasive exotic tree that has dramatically increased in abundance in its' non-native range.

An invasive plant species of particular concern is the deciduous Chinese tallow tree (*Triadica sebifera*, Fig. 1), native to China and Japan (Bruce et al. 1997). This tree species, with massive invasive potential, is known to replace native vegetation and produce monocultures particularly in wetland habitats (Cameron and Spencer 1989; Jubinsky and Anderson 1996; Bruce et al. 1997). The current invasive distribution of Chinese tallow includes much of the South Atlantic and Gulf Coastal Plain (Conner et al. 2002), and it is now the fifth most numerous tree species in the entire state of Louisiana and the fifth most common species found in east Texas (Oswalt 2010).

Recent studies have shown that exposure to Chinese tallow leaf litter has negative effects on tadpole survival compared with leaf litter of native tree species; however, the relative impact on survival varied among amphibian species (Leonard 2008). Variable survival rates were also seen among four anuran species in east Texas when exposed to submerged Chinese tallow leaf litter (Cotten et al. 2012). Two winter breeding species, the southern leopard frog (*Lithobates sphenocephalus*), and Cajun chorus frog (*Pseudacris fouquettei)* had lower survival rates when exposed to Chinese tallow leaf litter compared with native leaf litter, while spring and summer breeding anuran species did not experience lower survival in Chinese tallow treatments (Cotten et al. 2012). Cotten et al. (2012) suggested that the negative effects of Chinese tallow leaf litter seen on winter breeding species, but not in the summer breeders, could be due to short-lived effects from the rapid breakdown of Chinese tallow leaves. The implication of the Cotten et al. (2012) supposition is that timing of amphibian breeding and leaf fall may be factors critical to the relative impact of invasive Chinese tallow on amphibian survival. Given that amphibian breeding phenology is highly correlated with local weather conditions (Saenz et al. 2006), and Chinese tallow leaf fall can be influenced by freezing temperatures (Cameron and Spencer 1989), there is potential for climate change to indirectly influence native amphibian survival in the presence of invasive plants by altering the timing of amphibian breeding and leaf fall. Mistiming of amphibian and plant phenologies could induce amphibian breeding to occur when the negative effects of the invasive plants are the greatest. This is of particular concern, given that climate models project a 2-10^o^ C increase in temperature by the end of this century in the southeastern United States, the current range of invasive Chinese tallow (IPCC 2007).

Yang and Rudolf (2010) suggest that some of the most profound effects of climate change are likely to be caused by changes in the timing of biotic interactions between species. The mistiming of species interactions caused by climate change is well documented (Parmesan 2006) as is the evidence for reduced fitness cause by the asynchrony (Visser and Both 2005). Of the mistimed interactions, predator–prey interactions appear to be most common but insect–host interactions and competitor–competitor interactions also have been studied (Visser and Both 2005; Both et al. 2009; Rudolf and Singh 2013).

In this study, we attempt to elucidate how changes in climate can affect the interaction among invasive plant species and native anurans. To accomplish this goal, we examined the following objectives: (1) determine differences in survival and morphology of anuran larvae when exposed to treatments containing water and Chinese tallow leaf litter in five different stages of decomposition. The various treatments are intended to simulate differences in anuran breeding and Chinese tallow leaf phenologies that are influenced by different climate scenarios. (2) determine whether there are differences in water chemistry among treatments (pH, salinity, and dissolved oxygen) containing water and Chinese tallow leaf litter in different stages of decomposition. (3) determine whether water chemistry variables are associated with survival and morphology of anurans exposed to Chinese tallow leaf litter.

## Material and Methods

### Anuran species

We chose the southern leopard frog as our focal amphibian species for this study because it is widespread throughout the southeastern United States, making it sympatric with Chinese tallow in nearly all of the plant's invasive range (Conant and Collins 1998). In addition, the southern leopard frog is considered a biannual breeder throughout much of its range, breeding in fall and spring after ephemeral ponds have been filled with rain (Ryan and Winne 2001). In east Texas, southern leopard frogs have been observed calling every month of the year, with peaks in spring and fall that are dependent upon temperature and precipitation (see Saenz et al. 2006 for details about southern leopard frog calling phenology and relationships between calling activity and weather variables). In addition to calling data, a small number of published studies have reported on the collection of southern leopard frog eggs in the field in eastern Texas. Johnson et al. (2003), Cotten et al. (2012), Adams and Saenz (2012), and this study all report colleting eggs in late January, while Saenz et al. (2003) collected eggs in December. In an unpublished study in eastern Texas, Saenz et al. (unpubl. data), found southern leopard frog eggs in February, March, April, and October, providing further evidence that this anuran species will reproduce any time of year, provided suitable weather conditions. Given the southern leopard frog's breeding habitat preferences and breeding phenology, this species is very likely to be exposed to Chinese tallow leaf litter in nature.

### Chinese tallow

In addition to amphibian breeding phenology, an understanding of Chinese tallow leaf fall phenology is critical to this study. Cameron and Spencer (1989) provide a detailed multiyear account of leaf fall in a Chinese tallow monoculture in eastern Texas. Using leaf traps, they determined that leaves typically started to fall in October and continued into February in some years. Most of the leaf fall in their study took place in November followed by December; however, the timing of leaf fall was greatly influenced by weather, as leaf fall increased substantially after the first hard freeze of the season. The general phenology and the observed plasticity of the timing of leaf fall in Chinese tallow suggests that the overlap in the timing of the leaf litter in wetlands and anuran breeding will vary from year to year, largely dependent on weather.

### Leaf collection

Chinese tallow leaves were collected from the Stephen F. Austin State University campus, the Stephen F. Austin Experimental Forest (SFAEF), and around the cities of Nacogdoches, Lufkin, and Huntington, Texas, during the first 2 weeks of November 2009. Senesced leaves that had changed color from green to reds and oranges were stripped from cut trees or freshly fallen leaves were collected. Leaves were air-dried in a dark climate-controlled room and stored in black plastic bags until used in experiments. Prior to this study, we conducted laboratory trials to compare the “potency” of Chinese tallow leaves that had been stored for over a year to newly collected leaves by submerging standardized concentrations of leaves in containers and measuring changes to water chemistry. We found no differences in water chemistry readings between year-old and newer leaf trials; therefore, we felt confident that storing leaves for a maximum of 12 weeks would not be problematic for this study.

### Mesocosm set-up

One hundred mesocosms were set up on a site approximately 13.7 × 18.3 m within the SFAEF using 100-L blue plastic wading pools. All pools were exposed to the same light cycle, weather conditions, and temperature. Each pool was filled with 80 L of well water from the Stephen F. Austin Experimental Forest. Approximately 150 g of dried Chinese tallow leaf litter were added to the water to produce roughly 1.875 g of leaf litter per liter of water (Cotten et al. 2012). Four 9.5-mm holes were drilled on the sides of each pool just above the water line to prevent material from spilling over during heavy rainfall. Mesocosms were placed in rows at the SFAEF and were covered with 30% shade cloth to prevent invertebrate predators and other amphibians from entering the mesocosms.

### Treatments

The experimental mesocosms were randomly assigned to one of five different treatments representing different amphibian breeding phenology and leaf decomposition scenarios that could be realized through different weather conditions and climate change. Each treatment was composed of 20 mesocosms with Chinese tallow leaf litter at various stages of decomposition. The first treatment (T1) was set up on 24 November 2009, nearly 10 weeks before southern leopard frog tadpoles were introduced. The scenario created by T1 was intended to simulate a condition where amphibians breed later in the year and Chinese tallow leaf fall occurred earlier in the season, perhaps because of lower temperatures, allowing the leaf litter many weeks to decompose before the arrival of breeding amphibians. The subsequent three treatments (T2–T4) were set up at approximately 2-week intervals following T1. The last treatment (T5) was installed on 22 January 2010, just 8 days prior to the introduction of tadpoles. The scenario created by T5 would represent a case where amphibian breeding phenology is shifted to an earlier date or by Chinese tallow trees holding leaves later into the winter, which is likely in the event of a warming climate, as suggested by Parmesan (2007) and Cameron and Spencer (1989), respectively. The earlier breeding suggested by T5 would expose amphibian larvae to Chinese leaf litter that has had a short time to decompose, because the timing of breeding will take place closer to natural leaf fall for the invasive tree. Treatments T2–T4 were intended to be intermediates between the extremes of the first and last treatments.

### Water chemistry measurements

We used a Quanta^©^ Hydrolab Water Quality Monitoring System to measure pH (Hydrolab Corporation, Austin, TX), salinity (PSS), and dissolved oxygen (DO, mg/L), once per week. Water chemistry measurements began when T1 was installed, 24 November 2009, and only took place in mesocosms with leaf litter material in them. For example, during the first 2 weeks of the experiment, mesocosms in T1 were the only mesocosms present to be measured. After 2 weeks, T2 was installed; therefore, mesocosms from T2 were measured along with mesocosms from T1. The number of mesocosms measured weekly increased as each new treatment was installed until all five treatments (100 mesocosms) were present. Once tadpoles were removed, water chemistry measurements were taken every 2 weeks until 4 May 2010.

### Anuran egg collection and tadpole introduction

Twenty southern leopard frog egg masses were collected from the Stephen F. Austin Experimental Forest on 20 January 2010. The eggs were hatched and allowed to develop to Gosner stage 25 (Gosner 1960) to ensure that larvae were healthy prior to introduction into the mesocosms. Tadpoles were introduced into all five treatments on 30 January 2010. There were 20 mesocosms per treatment for a total of 100 mesocosms. Each mesocosm contained only 20 tadpoles that were full siblings, from the same clutch. Tadpoles from each of the 20 clutches were randomly assigned to a mesocosm in each treatment. Hence, each egg mass was represented once in each treatment with 20 individuals per mesocosm.

Once tadpole development approached Gosner stage 40 in any individual (Gosner 1960), all tadpoles were pulled from the treatments to prevent metamorphs from dispersing. Tadpoles were retrieved on 14 April 2010, 75 days after being introduced into the treatments. Tadpoles from each mesocosm were counted to determine the number surviving from the original 20. Remaining tadpoles were euthanized using MS222 and preserved in 10% formalin. Developmental stage (Gosner 1960) was determined for all tadpoles and head length, tail length, tail depth, tail muscle depth, and total length were measured using digital calipers. Measurements were taken to the nearest 0.01 mm.

### Analysis

Maternal effects can also influence growth and metamorphosis in tadpoles (Loman 2002) and potentially act as confounding variables. To control for maternal effects, differences in all measurements between clutch means (*n* = 5 mesocosms/clutch, 1 from each treatment) and mesocosm means (within clutches) were calculated for each morphological variable. This produced a deviation from the overall clutch mean for every mesocosm. Deviations were then added to the mesocosm morphometric means to produce values that express treatment effects but hold clutch effects constant (Cotten et al. 2012). These values were used for the subsequent analyses.

Pairwise permutation tests were used to compare differences in morphology and survival of tadpoles among the five treatments. A permutation test creates a distribution of possible outcomes by reshuffling and sampling one's own data (Adams and Anthony 1996). *PopTools* 3.0.5 for Microsoft Excel was used to perform the permutation test. Pairwise treatment differences were calculated from adjusted values (controlled for maternal effects) by taking the absolute difference between treatment means. Adjusted mesocosm values were then randomly re-assigned (without replacement) to treatments, then another absolute difference, using the resampled data, was calculated. By performing this procedure many times, a distribution of possible differences was generated (Adams and Anthony 1996). We used 10,000 iterations with the original adjusted values counting as one iteration. The absolute difference from the observed values was compared with the distribution of absolute differences of the resampled values to determine the likelihood that value observed in the original adjusted data could be found by chance (Adams and Anthony 1996). The *P*-value for the test was calculated as the proportion of iterations where the absolute difference in randomized treatment means was greater than, or equal to, observed adjusted treatment mean differences. A false discovery rate (FDR) adjustment was performed on the multiple tests (Benjamini and Hochberg 1995). Observed values were considered significant if the adjusted *P*-value was < 0.05.

Pairwise permutation tests were also used to compare differences in pH, salinity, and DO among the five treatments. Mesocosm means were adjusted as described above for the survival and morphology comparisons. Because changes in water chemistry happen soon after submerging leaf litter in water, we compared measurements from the first three dates after leaves were placed in T5 (the last treatment to receive leaf litter). Water chemistry measurements from these 3 days also provided data before and after the introduction of tadpoles into treatments. The three dates tested were 26 January 2010 (4 days before the introduction of tadpoles), 2 February 2010 (3 days after tadpoles were placed in mesocosms), and 9 February 2010 (9 days after tadpoles were placed in mesocosms).

## Results

### Survival

**Figure 2:**
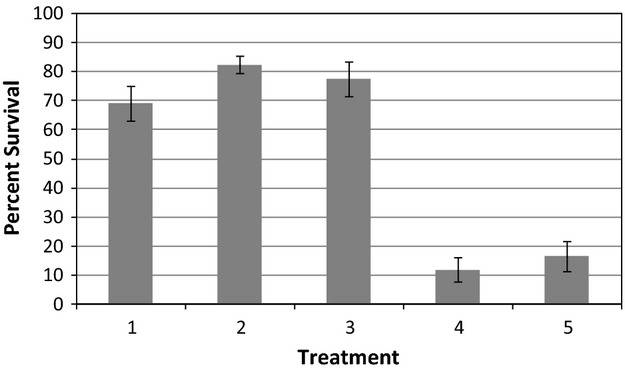
Mean percent survival for or southern leopard frog tadpoles exposed to Chinese tallow leaf litter at different stages of decomposition. Bars with similar letters did not differ (*P* ≥ 0.05); pairwise tests comparing the absolute difference between adjusted observed values and permuted values, followed by a FDR adjustment of the *P*-values. Error bars represent one standard error.

Survival differed significantly among treatments (Fig. 2). Tadpoles in treatments with Chinese tallow leaf litter submerged in water for the longest amount of time, T1, T2, and T3, had significantly higher tadpole survival than T4 and T5, the treatments that had tallow submerged for the shortest amount of time (*P* < 0.0001). Treatments T4 and T5 had a combined survival rate that was over five times lower than the combined rate of the first three treatments (Fig. 2). Survival was determined only at the end of the 75-day period, so it is unclear at what point mortality occurred within mesocosms.

### Morphology

Tadpoles did not differ (*P* ≥ 0.1278) in tail length, total length, and head length among treatments. Mean tail length for the five treatments ranged from 23.2 to 25.0 mm, total length ranged from 33.0 to 35.5 mm, and head length ranged from 13.1 to 14.1 mm.

Mean tail muscle depth and tail depth were the only morphological variables that differed among the five treatments. Mean tail depth for the five treatments ranged between 8.6 and 7.9 mm. Tadpoles in treatments T1–T3 had significantly greater tail muscle depth than tadpoles in T5 and tadpoles in T2 had significantly greater overall tail depth than tadpoles in T5 (Table 1), indicating possible lower body condition in the last treatment. Average Gosner stage for the five treatments ranged between 27.4 and 28.8. Our results indicated that tadpole development in T1 and T3 was significantly different, although there did not appear to be an overall trend related to development rate and the length of time tallow leaves were submerged in water.

**Table 1:** Adjusted observed morphological values (*n* = 20 mesocosms/treatment) of southern leopard frog tadpoles exposed to different Chinese tallow leaf litter in different stages of decomposition

Morphology	Treatment									

	T1 (68) 1		T2 (54)		T3 (40)		T4 (28)		T5 (8)	
				
	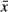	SE	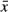	SE	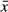	SE	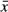	SE	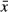	SE
Tail muscle depth (mm)	3.4 A		3.4 A		3.4 A		3.3 AB		3.0 B	
Tail depth (mm)	8.4 AB^2^		8.4 A		8.6 AB		8.5 AB		7.9 B	
Gosner stage	28.7 A		27.8 AB		28.1 B		28.2 AB		28.1 AB	

1 Number of days tallow leaf litter was submerged in mesocosms prior to the addition of tadpoles.

2 Values in a row with similar letters did not differ (*P* ≥ 0.05); pairwise tests comparing the absolute difference between adjusted observed values and permuted values, followed by a FDR adjustment of the *P*-values.

### Water chemistry

**Figure 3:**
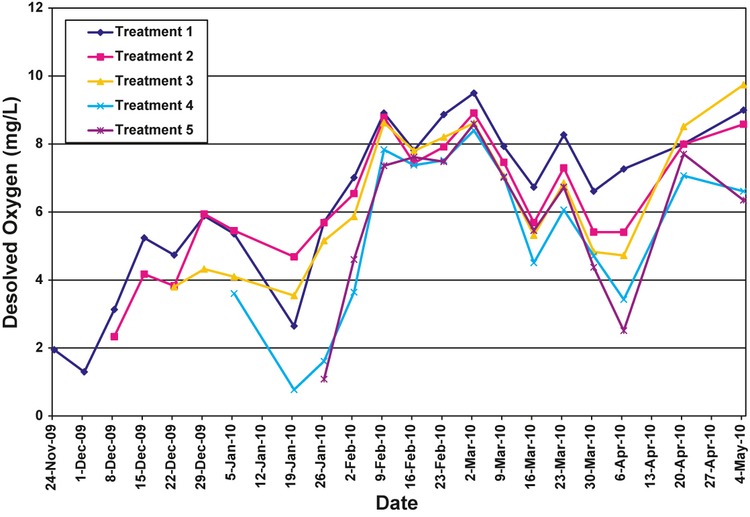
Timeline of dissolved oxygen (DO) measurements taken weekly from different levels of Chinese tallow leaf litter.

Dissolved oxygen differed significantly among treatments during the three sampling periods just prior to and after tadpole introduction (Table 2 and Fig. 3). In general, treatments T4 and T5, in which leaf litter was submerged in water for the shortest amount of time, had significantly lower DO than the three oldest treatments (Table 2). On 26 January, 4 days prior to tadpole introduction, mean DO ranged from 1.61 mg/L to 1.08 mg/L in treatments T4 and T5, respectively, which was roughly a fourfold difference in oxygen compared with the older three treatments (Fig. 3).

**Table 2:** Observed values of water chemistry measurements of different levels of Chinese tallow leaf litter made on three different dates (*n* = 20 mesocosms/treatment)

		Treatment									
		
		T1 (68)^2^		T2 (54)		T3 (40)		T4 (28)		T5 (8)	
						
Water chemistry	Time^1^	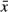	SE	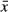	SE	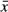	SE	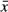	SE	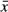	SE
Dissolved oxygen (mg/L)	4 days before	5.7 A^3^		5.8 A		5.1 A		1.7 B		1.1 B	
	3 days after	7.0 A		6.5 AB		5.9 B		3.7 C		4.6 D	
	9 days after	8.9 A		8.8 A		8.7 A		7.8 B		7.3 C	
pH	4 days before	6.59 A		6.63 A		6.72 B		6.82 C		6.78 BC	
	3 days after	6.56 A		6.57 AB		6.66 B		6.83 C		6.80 C	
	9 days after	6.66 A		6.68 A		6.77 B		6.88 C		6.89 C	
Salinity (PSS)	4 days before	0.06 A		0.07 A		0.09 A		0.17 B		0.19 B	
	3 days after	0.04 A		0.05 A		0.07 A		0.13 B		0.14 B	
	9 days after	0.03 A		0.03 A		0.04 A		0.08 B		0.09 B	

1 Number of days before or after the addition of tadpoles.

2 Number of days tallow leaf litter was submerged in mesocosms prior to the addition of tadpoles.

3 Values in a row with similar letters did not differ (*P* ≥ 0.05); pairwise tests comparing the absolute difference between adjusted observed values and permuted values, followed by a FDR adjustment of the *P*-values.

**Figure 4:**
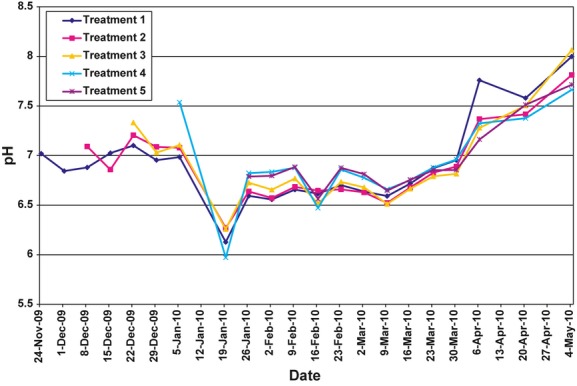
Timeline of pH measurements taken weekly from different levels of Chinese tallow leaf litter.

There were also significant differences in pH among treatments during our sampling periods that took place around the time of tadpole introduction (Table 2 and Fig. 4). The general trend was that the newer treatments, the ones which had tallow leaf litter submerged for the shortest amount of time, tended to have higher pH levels than the older treatments (Fig. 4); however, the differences in pH among the treatments were small, and all of the pH values were within the range that are normally seen in naturally occurring wetlands in east Texas (Saenz unpublished data).

**Figure 5:**
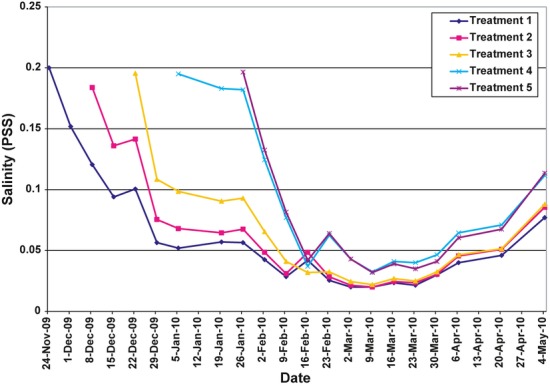
Timeline of salinity measurements taken weekly from different levels of Chinese tallow leaf litter.

Salinity was also found to differ significantly among treatments (Table 2 and Fig. 5). The last two treatments, in which tallow was submerged in water for the shortest amount of time, had significantly higher salinity than the three earlier treatments (Table 2). Salinity levels, in all treatments were not abnormally high and were well within the range of salinity levels observed in naturally occurring wetlands in east Texas (Saenz unpublished data).

### Timeline

**Figure 6:**
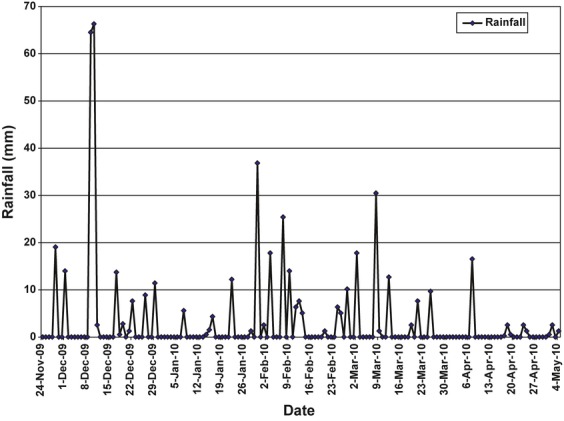
Timeline of daily rainfall measurements taken at the Stephen F. Austin Experiment Forest.

Water chemistry variables were measured 24 times, from 24 November 2009 to 4 May 2010, which yielded insight into changes that can occur in wetlands over time. We observed a general trend that indicated that DO was quickly lowered by the introduction of Chinese tallow leaf litter into water, then increased over time; however, there was much variation between sampling periods (Fig. 3). We found a very similar trend of increasing pH over time, with some variation between sampling bouts (Fig. 4). The opposite trend was observed for salinity. We saw a general decrease in salinity throughout most of the study; however, salinity levels began to rise over the last several sampling periods (Fig. 5), possibly due to lack of rain and evaporation. Throughout the duration of the study, we had approximately 40 rain events at the study site (Fig. 6). Some of the variation in the observed water chemistry variables could be related to rain events and extended periods of no rain.

## Discussion

Results of this study support the hypothesis that, by altering anuran breeding and or Chinese tallow leaf fall phenology, climate change could negatively influence the timing of the interaction between invasive Chinese tallow and native amphibians. We found tadpole survival to be lowest when exposed to Chinese tallow leaf litter that was submerged in water for the shortest amount of time. Low survival of southern leopard frog tadpoles could have been caused directly by temporary effects of toxic tannins leached from leaves or by short-term changes in water chemistry due to the chemical and physical breakdown of leaves and associated microbial activity (Adams and Saenz 2012).

Toxic effects of tannins on aquatic organisms can happen soon after leaves are immersed in water (Tremolieres 1988). Maerz et al. (2005b) showed that tannins leached from non-native purple loosestrife directly affected American toad (*Anaxyrus americanus*) tadpoles, causing rapid mortality compared with native broad-leaf cattail (*Typha latifolia*) extract. Most mortality of *A. americanus* occurred within 7 days of exposure to *L. salicaria* extract (Maerz et al. 2005b). Tannins in bark extract released in the debarking process at paper mills have been shown to cause epithelial damage on carp (*Cyprinus carpio)* gills (Temmink et al. 1989).

Tadpole survival could also be affected by deoxygenation of water due to chemical and physical breakdown of leaves and biological oxygen demand of microbial communities. Changes in oxygen concentrations can affect development, growth, activity, reproductive capacity, and survival of a variety of organisms (Bradford 1983; Moore and Townsend 1998). Studies show that under hypoxic conditions, tadpoles will swim to the surface for air (Wassersug and Seibert 1975; Moore and Townsend 1998). Wassersug and Seibert (1975) showed that *L. pipiens* tadpoles between Gosner stages 37 and 43 did not swim to the surface until DO levels were below 3 mg/L and at 2 mg/L a higher number of individuals were swimming to the surface. At DO concentrations of 2.0 mg/L *Bufo woodhousii* tadpoles lay motionless on their backs and sides and in one case a stage 41 *B. woodhousii* tadpole died at DO levels of 1.9 mg/L, of presumed hypoxia (Wassersug and Seibert 1975). Based on field observations, *Lithobates* tadpoles will gulp air at early stages of development if water is deficient in DO (Noland and Ultsch 1981). In addition to direct effects of hypoxia, there is an increased risk of predation for anuran tadpoles when abiotic variables change (Moore and Townsend 1998). Low DO levels can also alter development or kill amphibian embryos (Adams and Saenz 2012; Seymour and Bradford 1995). In our study, treatments with Chinese tallow leaf litter submerged for the shortest amount of time, <4 weeks, had DO levels below 1.7 mg/L. We suggest that the temporary hypoxia, induced by Chinese tallow leaf litter decomposition; will have direct negative effects on survival of amphibians and possibly indirect effects on behavior and predator defense.

During the initial step in leaf breakdown, when a leaf senesces and falls into a body of water, there is rapid loss of soluble organic and inorganic material due to leaching (Webster and Benfield 1986). According to previous studies on other leaf species, litter breakdown occurs in two phases, both of which cause deoxygenation of water (Tremolieres 1988; Canhoto and Laranjeira 2007). The first phase of deoxygenation happens within a few minutes and is caused by endogenous enzymatic oxidation of polyphenolic leaf compounds, such as tannins (Tremolieres 1988; Canhoto and Laranjeira 2007). The second phase of deoxygenation is due to oxygen demand of large fungal and bacterial communities (Tremolieres 1988; Canhoto and Laranjeira 2007). Also, slower oxidative processes, related to the formation of phytomelanic oxidative polycondensates, occur within 3–6 weeks and could contribute to long-term depletion of water oxygen (Tremolieres 1988).

In a laboratory study, deoxygenation was most evident soon after the initial leaching and breakdown process of tallow leaves immersed in water. Chinese tallow leaves immersed in water at concentrations of 2 g/L caused DO levels to drop drastically within 48 h, falling to approximately 1.1 mg/L compared with 8 mg/L in controls (Adams and Saenz 2012); however, lower DO was shown to rebound to higher levels over time. Adams and Saenz (2012) discovered that the concentration of Chinese tallow leaf litter in water negatively correlated with short-term DO levels. They also found that DO levels rebound more slowly with higher concentrations of Chinese tallow leaf litter, suggesting that the length of time tallow leaf litter might be harmful to amphibians is relative to tallow concentration; the higher the concentration the longer DO levels remain low.

Salinity and pH levels during this study were similar to levels seen in naturally occurring wetlands. During the time tadpoles were in treatments, pH levels were between 6.4 and 7.7. Measurements taken from over 50 ponds in east Texas revealed pH levels ranging from 5.5 to 6.9 (Saenz, unpubl. data). Dale et al. (1985b) demonstrated that *L. sylvatica* larvae maintained in water with a pH of 4.0 for 30 days appeared to have normal swimming and feeding behavior compared with controls. *Lithobates clamitan*s are also known from sites in Nova Scotia that had pH levels of 3.9 (Dale et al. 1985a). *Lithobates tigrina* exhibited erratic, jerky movements when exposed to ponds with pH <4.5 or higher than 8.5 and 50% of mortality occurred after 96 hours of exposure to pH of 9.5 (Abbasi et al. 1989). Adams and Saenz (2012) did not test the upper limits of pH tolerance in their study; however, *L. sphenocephalus* eggs did not survive at pH levels below 5.29, but hatched normally at pH levels up to approximately 7.0. Based on available literature and field observation, it seems unlikely that the range of pH levels found in this study had a negative influence on tadpole survival.

Salinity levels in our study ranged from 0.05 to 0.2 PSS at the time of tadpole introduction. The mean salinity level in wetlands sampled in east Texas was approximately 2.6 PSS (Saenz, unpubl. data). Christman (1974) found that *L. sphenocephalus* tadpoles, taken from freshwater habitats in Florida, could tolerate water with salinity up to 10.8 PSS. Five species of tadpoles were sampled in Victoria, Australia, wetlands with average salinity levels of approximately 2.1 PSS (Smith et al. 2007). Chinathamby et al. (2006) also found that the brown tree frog (*Litoria ewingii)* did not show reduced survival or decreased size at metamorphosis until salinity levels were approximately 5.6 PSS. Salinity levels during this study never exceeded 0.2 PSS. Therefore, differences in salinity levels in our study likely did not affect survival or morphology differences among our treatments.

In nature, the effects of leaf litter decomposition may be mediated by weather. We suspect that some of the drastic variation in DO that we observed in our study, between weekly sampling periods, was associated with rain events that increased DO. This would be consistent with research that indicates that the direct addition of oxygen from oxygen-saturated raindrops can be an important factor in reaeration in some wetlands (Banks and Herrera 1977; Ho et al. 1997). Rainfall events, soon after leaf fall, could mitigate the loss of oxygen caused by freshly submerged Chinese tallow leaf litter in natural systems, reducing the impacts on native fauna. The size of the wetland can play a role as well, as small ponds might be at greater risk of incurring higher concentrations of leaf litter, simply due to low volumes of water. Higher concentrations of tallow leaf litter are likely to induce lower DO levels that persist for longer periods of time (Adams and Saenz 2012). In addition, amount of surface area of a wetland may be critical to oxygen diffusion at the water/air interface. Greater surface area of larger ponds should facilitate reoxygenation compared to small wetlands with less surface area. Like DO, pH and salinity levels appeared to be affected by rain and evaporation and are likely influenced by local soil chemistry and other naturally occurring buffers.

Although weather may be a factor influencing water chemistry, we suggest that weather may play a greater role in the survival of amphibians in the presence of Chinese tallow leaf litter by regulating the relative timing of amphibian breeding and leaf fall. Summer breeding amphibians should not come into contact with Chinese tallow leaf litter until much of the decomposition process is over, so there should be little or no effect on survival or morphology from leaf litter decomposition, as was seen in the Cotten et al. (2012) study. Negative effects of Chinese tallow will likely be limited to winter breeding amphibians and amphibians that overwinter as larvae because they are more likely to be exposed to freshly leached tallow leaves and associated changes to water chemistry. Annual variation in winter weather, temperature and to a lesser degree rainfall, will determine amphibian breeding phenology (Saenz et al. 2006) and determine when Chinese tallow leaves fall and begin to decompose in water.

Although an infinite number of weather scenarios exist that could lead to variable responses in amphibian survival in the presence of Chinese tallow, we can make generalizations based on our findings. For example, wet weather in early winter will initiate the leaching and decomposition of tallow leaves that can impact amphibian survival. Early dry conditions will normally not allow breeding in ephemeral wetlands, which will cause delays in breeding. In general, a colder winter will delay anuran breeding and may expedite Chinese tallow leaf fall, regardless of precipitation. A warm wet winter may be the worst case scenario because it will induce early amphibian breeding, soon after leaf fall, in ephemeral wetlands that are more likely to have higher concentrations of Chinese tallow leaf litter. This could result in amphibian eggs entering and larvae developing in wetlands at a time when they would be exposed to hypoxic and tannic aquatic conditions, which could ultimately lead to lower survival.

It is unclear how climate change might affect the general phenology of Chinese tallow leaf fall. Climate warming may allow the trees to hold leaves later into the winter (Penuelas et al. 2002; Menzel et al. 2006), making overlap between leaf fall and amphibian presence more likely. Geographical differences in weather, across the range of Chinese tallow, could also influence breeding phenology and interactions between amphibians and Chinese tallow. Chinese tallow has the potential to spread throughout the southeastern United States and as global temperatures increase, the range of Chinese tallow could also increase to more northern areas (Pattison and Mack 2008, 2009; Gan et al. 2009; Wang et al. 2011).

As it becomes clearer that changes to water chemistry is a primary mechanism by which invasive plants reduce survival of aquatic native amphibians (Maerz et al. 2005b; Watling et al. 2011; Adams and Saenz 2012), this study indicates that amphibian breeding phenology and timing of leaf fall will ultimately determine the impact of Chinese tallow. Local weather is an important driver of amphibian breeding and Chinese tallow leaf fall phenology (Cameron and Spencer 1989; Oseen and Wassersug 2002; Saenz et al. 2006), and it will likely have influence on the interaction between the invasive Chinese tallow and native anurans on a year-to-year basis. Climate change, however, could mean larger and longer term shifts in phenology that could place aquatic amphibians at a consistent state of great risk to the deleterious effects of Chinese tallow.
